# Bioanalytical Method for Carbocisteine in Human Plasma by Using LC-MS/MS: A Pharmacokinetic Application

**DOI:** 10.3797/scipharm.1403-12

**Published:** 2014-05-22

**Authors:** Shivanand Dhanure, Atulkumar Savalia, Pravinkumar More, Prashant Shirode, Kailas Kapse, Virag Shah

**Keywords:** Carbocisteine, Bioanalytical, Plasma, Chromatography, Rosiglitazone, LC-MS/MS

## Abstract

A simple, sensitive, and selective LC-MS/MS method was developed and validated for the quantification of carbocisteine in human plasma. Rosiglitazone was used as the internal standard and heparin was used as the anticoagulant. The chromatographic separation was performed by using the Waters Symmetry Shield RP 8, 150 × 3.9 mm, 5 μ column at 40°C with a mobile phase consisting of a mixture of methanol and 0.5% formic acid solution in a 40:60 proportion. The flow rate was 500 μl/min along with a 5 μl injection volume. Protein precipitation was used as the extraction method. Mass spectrometric data were detected in positive ion mode. The MRM mode of the ions for carbocisteine was 180.0 > 89.0 and for rosiglitazone it was 238.1 > 135.1. The method was validated in the concentration curve range of 50.000 ng/mL to 6000.000 ng/mL. The retention times of carbocisteine and the internal standard rosiglitazone were approximately 2.20 and 3.01 min, respectively. The overall run time was 4.50 min. This method was found suitable to analyze human plasma samples for the application in pharmacokinetic and BA/BE studies.

## Introduction

Carbocisteine (*S*-carboxymethylcysteine or SCMC) is a mucoactive drug with *in vitro* free-radical scavenging and anti-inflammatory properties. It was first synthesized in 1930 and initially used as a mucoregulator. Due to the lack of consistency in data on clinical efficiency in the UK, the National Health System (NHS) formulary blacklisted carbocisteine in the 1980s. However, as per new data of several trials demonstrating the positive effects of mucoactive drugs, the importance of exacerbation frequency in the progression of chronic obstructive pulmonary disease (COPD) and the role of systemic inflammation in the disease process have rekindled interest in carbocisteine. Once again, it became available in the NHS prescription for chronic use in COPD in 2003 [1].

The oral preparation of carbocisteine is available as SCMC and its lysine salt (SCMC-Lys). The active drug SCMC is formed by the cleavage of the lysine group during gastric absorption. Carbocisteine is a blocked thiol derivative of the amino-acid, L-cysteine. The mechanism of action of carbocisteine is different than other commonly available mucolytic drugs such as N-acetylcysteine (NAC) and erdosteine that bear free sulfhydryl (thiol) groups via which they split glycoprotein bonds in mucus [1].

Chemically, carbocisteine is (*R*)-2-amino-3-(carboxymethylsulfanyl)propanoic acid with the molecular formula C_5_H_9_NO_4_S and molecular weight of 179.19 g/mol [2].

It was found that very little literature was available on the bioanalytical methods for the determination of carbocisteine in the human matrix i.e. plasma, urine. Available analytical methods for carbocisteine were by HPLC determination with UV detection; however, these methods had many disadvantage like less sensitivity, high injection volume, etc. [3–8].

In view of the above discussion, the authors have decided to develop a newer bioanalytical method for carbocisteine by using LC-MS/MS.

## Experimental

### Materials and Reagents

**Fig. 1.**
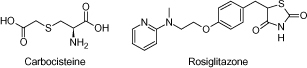
Chemical structures of carbocisteine and rosiglitazone

The purity of the carbocisteine working standard, supplied by Micro Labs Limited, India, was 99.0% and the purity of the rosiglitazone working standard, supplied by Neucon Pharma Pvt. Ltd., India was 94.75%, which was used as the internal standard. The chemical structure of both of the compounds is presented in Figure 1. HPLC grade organic solvents like formic acid (Sigma Aldrich, USA) and methanol were used for analysis (J.T Baker, USA). Milli-Q water used for analysis was prepared from the Millipore System (Bangalore, India). The human plasma containing heparin as the anticoagulant was procured from Cauvery Diagnostics and Blood Bank (Secunderabad, India).

### Chromatography

Chromatography separation was carried out by using the Symmetry Shield RP8, 150 × 3.9 mm, 5 μm. The retention times of carbocisteine and rosiglitazone were approximately 2.20 and 3.01 min, respectively. The overall run time was 4.50 min. An HPLC system of Shimadzu (Japan) containing an autosampler (SIL-HTc), pumps (LC-20AD), column oven (CTO-10AS VP), and degasser (DGU-20A3) was used for the separation of the analytes.

The injection volume was 5 μL at the autosampler temperature of 10°C. The mobile phase consisting of methanol: 0.5% and formic acid (40:60, v/v) was used for the analysis.

### Mass Spectrometer Conditions

Quantitation was done by using MS–MS detection in the Applied Biosystems API–3200 mass spectrometer (Foster City, CA, USA) equipped with a Turboionspray™ interface. The data were summarized by using Analyst 1.4.1 software. The detection was performed on positive mode ionisation along with ion spray, voltage 5500. A detailed summary of the mass parameters of carbocisteine and rosiglitazone are presented in Table 1.

**Tab. 1.** Summary of mass parameters of carbocisteine and rosiglitazone

Drug Name	Parent mass (Q1)	Parent mass (Q3)	Time (msec.)	DP(eV)	EP(eV)	CEP(eV)	CE(eV)	CXP(eV)
Carbocisteine	180.0	89.0	400	35	7	12	23	2
Rosiglitazone	358.1	135.1	300	55	8	13	36	2

### Preparation of the Calibration Curve and Quality Control Standards

Carbocisteine stock solution (1.000 mg/mL) was prepared in methanol. This stock solution was further diluted in the ratio of methanol: water (50:50). The concentration of working dilutions were 1.000, 2.000, 6.000, 20.000, 50.000, 80.000, 100.000, 120.000 μg/mL The 5% calibration curve and quality control working dilution spiking was done in blank human plasma containing heparin as an anticoagulant. The concentrations of the spiked plasma calibration curve were 50.000, 100.000, 300.000, 1000.000, 2500.000, 4000.000, 5000.000, 6000.000 ng/mL. The concentrations of the quality control working dilution samples were 3.000, 48.000, 90.000 μg/mL. The concentrations of the spiked plasma quality control samples were 150.000, 2400.000, 4500.000 ng/mL. All spiked plasma samples were stored in a deep freezer at -70±15°C.

Sample Treatment

An aliquot of 200 μL of a plasma sample was taken in 2-mL Eppendorf tubes. In it, 25 μL of the internal standard solution was added and vortexed for 20-30 seconds for complete mixing. Then 700 μL of methanol was added and again vortexed for 5 minutes. The prepared sample was centrifuged at 15,000 rpm for 5 min at 10°C. The 250 μL supernatant of the prepared sample was diluted with 250 μL of the dilution buffer solution (methanol and 0.5% formic acid, 20:80 v/v) and transferred into the HPLC vials for analysis.

### Method Validation

The method validation was performed by referring to USFDA guidelines for the industry [9]. The method validation parameters like blank matrix specificity, lower limit of quantification, injector carryover, precision and accuracy, recovery of the drug and internal standard, stability studies, dilution integrity, haemolysis effect, matrix effect, anticoagulant effect, re-injection reproducibility, and ruggedness were performed. In selectivity, randomly ten different blank human plasma matrix containing eight lots of heparin plasma (including one haemolysed plasma and one lipemic plasma) and two lots of plasma with a different anticoagulant (EDTA) were analyzed.

The lower limit of quantitation (LLOQ), i.e. the lowest standard level, was 50.000 ng/mL for carbocisteine. The signal-to-noise ratio of ten LLOQ samples obtained from the blank matrix specificity experiment were used for the lower limit of quantitation calculation. For injector carryover, one vial each for the resolution solution and mobile phase was prepared and processed with one sample LLOQ and upper limit of quantitation (ULOQ). The calibration curve range for carbocisteine was 50.000 ng/mL to 6000.000 ng/mL. The within-run accuracy, single-run precision, and accuracy batch were constituted with a calibration curve and six replicates of the LLOQ, lower quality control (LQC), middle quality control (MQC), higher quality control (HQC), and ULOQ samples. The between-run precision and accuracy batches were run on different occasions. Each run was constituted with a calibration curve and six replicates of the LLOQ (50.000 ng/mL), LQC (150.000 ng/mL), MQC (2400.000 ng/mL), and HQC (4500.000 ng/mL) samples.

The dilution integrity experiment was performed on 1.5 times the ULOQ working solution concentration to check the precision and accuracy of the samples after dilution.

The recovery of the analyte and internal standard was evaluated by comparing the bioanalytical results for the extracted QC samples with the equivalent unextracted low, medium, and high QCs.

The ruggedness experiment was performed to check the precision and accuracy of the samples when processed by a different analyst. The matrix effect was calculated by processing six blank matrices, including one sample each of the haemolysed and lipemic, as per the extraction procedure.

The matrix factor (MF) of the analyte was calculated by the ratio of the peak area in the presence of the matrix (measured by analysing a blank matrix spiked with the LQC and IS after extraction) to the average peak area in the absence of the matrix (pure solution of the analyte). Similarly, the MF of the internal standard (IS) was calculated. Also, the IS-normalised MF was calculated by dividing the MF of the analyte by the MF of the IS. The haemolysis effect was performed by processing six LQC and HQC samples spiked in the haemolysed plasma and analyzed it with freshly processed calibration standards in a single run. The anticoagulant effect was performed to check the precision and accuracy of the samples when processed in the matrix having a different anticoagulant (K_2_EDTA).

The stability of the analyte was performed in the stock solution and plasma samples. The different stability studies were performed i.e. short-term stability of the analyte in stock solution (16 h 15 min), short-term stability of the internal standard in stock solution (16 h 02 min), short-term stability of the analyte in working solution (16 h 40 min), short-term stability of the internal standard in stock solution (16 h 45 min), freeze-thaw stability (five freeze and thaw cycles), coolant stability (87 h 50 min), autosampler stability (72 h 20 min), benchtop stability (17 h 29 min), long-term stability of the analyte and internal standard in stock solution (25 days), long-term stability of the analyte and internal standard in working solution (24 days), and long-term stability in the biological matrix at -70±15°C and -20±10°C.

## Results and Discussion

### Blank Matrix Specificity

**Fig. 2.**
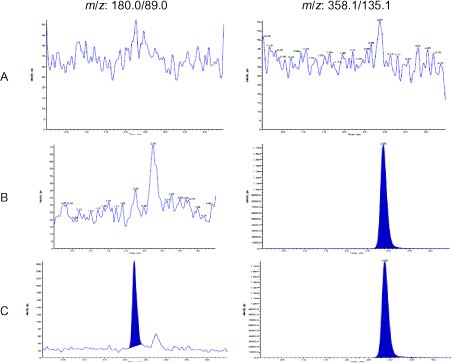
MRM chromatograms of carbocisteine (left side) and rosiglitazone (right side) in (A) human blank plasma; (B) human plasma spiked with IS; (C) an LLOQ sample along with IS

Blank human plasma containing heparin and ethylenediaminetetraacetic acid (EDTA) were extracted and analysed to determine the extent to which endogenous human plasma components may contribute to chromatographic interference with the analyte or the internal standard. No significant interferences were observed in eight different lots of human heparin plasma (including one lipemic and one haemolysed plasma) samples and two lots of human EDTA plasma samples. Figure 2 indicates the representative chromategrams: blank matrix, blank matrix with IS, and LLOQ.

### Evaluation of the Lower Limit of Quantification

The lower limit of quantitation must demonstrate a signal-to-noise ratio higher than or equal to 5.00. The lower limit of quantitation for carbocisteine was 50.069 ng/mL with a signal-to-noise ratio ≥ 5. The lower limits of quantitation were evaluated by comparing the peak height response of the extracted samples of the lowest calibration standard to the peak height response of the blank matrix samples.

### Injector Carryover

No significant injector carryover was observed.

### Calibration Curves and Regression Method

The calibration curves were found to be consistently accurate and precise over the calibration range of 50.069 ng/mL to 6008.310 ng/mL. The calibration curve of the area response was plotted with a “linear” fit and “1/x^2^” weighting. The correlation coefficient (r) was greater than or equal to 0.9990. The back-calculated calibration curve standards for the precision and accuracy batch (PA) are presented in Table 2 and a summary of the calibration curves are presented in Table 3.

**Tab. 2.** Back-calculated calibration curve standards for the precision and accuracy batch

Result Table ID	Nominal Concentration (ng/mL)							
	CS1	CS2	CS3	CS4	CS5	CS6	CS7	CS8
	50.069	100.139	300.416	1001.385	2503.463	4005.540	5006.925	6008.310
**PA Batch 01**	51.028	99.032	275.014	989.312	2576.289	3973.860	5083.477	6321.015
**PA Batch 02**	51.579	96.319	279.814	987.584	2560.405	3972.734	5158.012	6282.383
**PA Batch 03**	49.599	102.066	301.500	991.512	2486.624	3785.833	5002.643	6361.792
**PA Batch 04**	50.189	99.277	303.329	1012.377	2512.349	3835.860	5022.482	6135.991
**N**	4	4	4	4	4	4	4	4
**Mean**	50.599	99.174	289.914	995.196	2533.917	3892.072	5066.654	6275.295
**S.D.**	0.8780	2.3484	14.5856	11.5661	41.6285	95.9898	69.9473	98.3665
**% C.V.**	1.74	2.37	5.03	1.16	1.64	2.47	1.38	1.57
**% Nominal**	101.06	99.04	96.5	99.38	101.22	97.17	101.19	104.44

**Tab. 3.** Summary of the calibration curves

Result Table ID	Slope	Y-lntercept	Correlation Coefficient (r)
PA Batch 01	0.000395	0.001500	0.9990
PA Batch 02	0.000418	-0.000227	0.9990
PA Batch 03	0.000274	0.000831	0.9994
PA Batch 04	0.000305	0.000262	0.9998

### Limit of Quantitation

The lowest concentration for precision and accuracy was 50.069 ng/mL on the basis of the LLOQ determination. The lowest standard level was 50.069 ng/mL with a coefficient of variation of 3.35% and the nominal value was 99.88%.

#### Within-Run Accuracy and Precision

Analysis of the replicate concentrations of carbocisteine in human heparin plasma was performed for the evaluation of within-run accuracy and precision. The run consisted of a calibration curve plus a total of 30 spiked samples, six replicates each of the lower limit of quantification (LLOQ), upper limit of quantification (ULOQ), low, medium, and high quality control samples. The within-run coefficients of variation ranged between 1.70 to 3.62%. The within-run percentage of nominal values ranged from 96.26 to 100.51. Results are presented in Table 4.

**Tab. 4.** Within-run accuracy and precision

Concentration (ng/mL)	n	Mean	S.D.	% C.V.	% Accuracy
50.069	6	50.009	1.6757	3.35	99.88
150.609	6	147.034	4.6926	3.19	97.63
2409.739	6	2319.532	79.4949	3.43	96.26
4518.261	6	4441.881	160.9108	3.62	98.31
6008.310	6	6038.990	102.6913	1.70	100.51

### Between-Run Accuracy and Precision

The between-run accuracy and precision evaluations were assessed by the repeated analysis of human heparin plasma samples containing different concentrations of carbocisteine on separate occasions. A single run consisted of a calibration curve, six replicates each of the lower limit of quantification (LLOQ), low, medium, and high quality control samples. The between-run coefficients of variation ranged from 3.40 to 5.88%. The between-run percentage of nominal values ranged from 95.20 to 98.56. Results are presented in Table 5.

**Tab. 5.** Between-run accuracy and precision

Concentration (ng/mL)	n	Mean	S.D.	% C.V.	% Accuracy
50.069	24	49.347	2.9011	5.88	98.56
150.609	24	147.531	5.3771	3.64	97.96
2409.739	24	2303.909	78.2669	3.40	95.61
4518.261	24	4301.267	181.2429	4.21	95.20

### Recovery (Extraction Efficiency)

Recovery of carbocisteine was evaluated by comparing the mean analyte area response of six extracted samples of low, medium, and high quality control samples to the mean analyte area response of six un-extracted (samples prepared in the extracted plasma blank) samples of low, medium, and high quality control samples. For carbocisteine, the mean recovery values were 77.62%, 76.11%, 80.01% at the low, medium, and high quality control levels, respectively. Results are presented in Table 6. For the internal standard (rosiglitazone), the mean internal standard area response of six extracted low quality control samples were compared to the mean internal standard area response of six unextracted (samples prepared in the extracted plasma blank) samples at the low quality control level. The recovery value for the internal standard (rosiglitazone) was 83.33%.

**Tab. 6.** Recovery of the analyte from the biological matrix

%Recovery of LQC (150.609 ng/mL)	% Recovery of MQC (2409.739 ng/mL)	% Recovery of HQC (4518.261 ng/mL)	Average	Standard Deviation	% C.V.
77.62	76.11	80.01	77.91	1.9654	2.52

### Dilution Integrity

Six replicates of the diluted quality control (DQC) were respectively diluted to 1/5 and 1/2 in human heparin plasma prior to extraction and analysis. For carbocisteine, the calculated concentrations including the dilution factor for 1/5 and 1/2 yielded a coefficient of variation of 2.84 and 1.66%, respectively. The percentages of nominal values were 96.65 and 95.76, respectively.

### Reinjection Reproducibility

To evaluate the reinjection reproducibility experiment, the LQC and HQC samples of one precision and accuracy were kept in the autosampler after analysis at 10°C and reinjected after 2.00 h. Concentrations were calculated to determine the % change after reinjection. Carbocisteine was found to be stable in the autosampler at 10°C and reproducible after reinjection.

### Haemolysis Effect

Six samples each of the low and high QC were processed in the haemolysed blank plasma lot and analysed with the calibration (processed in blank human heparin plasma) set. Back-calculated concentrations were calculated to determine the % nominal. No effect of haemolysis was observed on the analytes’ quantitation. The coefficients of variation ranged from 1.67 to 2.76%. The nominal percentage ranged from 94.51 to 101.16.

### Anticoagulant Effect

Six samples each of low and high QC were processed in the EDTA blank plasma lot and analysed with the calibration (processed in blank human heparin plasma) set. Back-calculated concentrations were calculated to determine the % nominal. No effect of the anticoagulant was observed on the analytes’ quantitation. The coefficients of variation ranged from 2.91 to 3.75%. The nominal percentage ranged from 94.55 to 99.58.

### Matrix Effect

Processed post-extracted samples and aqueous samples (six injections of the aqueous sample) were analysed in a single run. No effect of the matrix was observed on the analytes’ quantitation. The matrix factor for carbocisteine and rosiglitazone was 0.84 and the internal standard normalized matrix factor was 1.01.

### Stability Tests

The stability tests were performed for stock solution stabilities and biological matrix stabilities. The stock solution stability is presented in Table 7. The concentrations used in the stability samples for the stock solution were 1.001 mg/mL for carbocistine and 197.101 μg/mL for the internal standard (rosiglitazone). The comparison stability sample concentrations were 1.004 mg/mL for carbocistine and 199.318 μg/mL for the internal standard (rosiglitazone).

Different biological matrix (plasma) stability tests were performed like benchtop stability, autosampler stability, freeze–thaw cycles, reinjection stability, and long-term stability at -70°C and -20°C for 23 days, the mean % nominal values of the analytes were found to be within ±15% of the predicted concentrations for the analytes at their LQC (150.609 ng/mL) and HQC (4518.261 gn/mL) levels (Table 8). Thus, the results were found to be within the acceptable limits during the entire validation.

**Tab. 7.** Summary of stock solution stability tests

Stability	Duration	% Change
Short-term stability of analyte in stock solution	16 h 15 min	-0.61
Short-term stability of internal standard in stock solution	16 h 02 min	-0.58
Short-term stability of analyte in working solution	16 h 40 min	-0.76
Short-term stability of internal standard in stock solution	16 h 45 min	-0.67
Long-term stability of analyte in stock solution	25 days	1.20
Long-term stability of internal standard in stock solution	25 days	1.47
Long-term stability of analyte in working solution	24 days	3.19
Long-term stability of internal standard in working solution	24 days	3.00

**Tab. 8.** Summary of biological matrix stability tests

Stability	Duration	% Change of LQC	% Change of MQC
Freeze-thaw stability	5 Cycles	-1.53	0.65
Coolant stability	87 h 50 min	1.52	0.98
Autosampler stability	72 h 20 min	-0.86	2.68
Benchtop stability	17 h 29 min	1.65	-0.75
Long-term stability in biological matrix at 20 ± 10°C	23 days	1.91	-3.92
Long-term stability in biological matrix at 20 ± 10°C	23 days	1.21	-2.15

### Incurred Sample Reanalysis

A total of 78 samples (5.06% of the total study samples) were selected for incurred sample analysis. One-hundred percent of the samples were within the specified acceptance limit, hence, it can be concluded that the LC-MS/MS method used for the analysis of the clinical study samples for carbocisteine is accurate, precise, and rugged and the data obtained by using this method can be used to evaluate pharmacokinetics from clinical studies.

### Pharmacokinetic Application

This validated method was applied for an open label, balanced, randomized, two-treatment, two period, two sequence, single dose, crossover bioavailability study comparing a carbocisteine capsule in 35 healthy, adult, human subjects under fasting conditions. In each period, a total of 22 blood samples (6 mL each) were collected. In the blood sampling, the first sample was collected within 1 hour prior to drug administration (0.0 hour) and subsequent samples were collected at 0.25, 0.50, 0.75, 1.00, 1.25, 1.50, 1.75, 2.00, 2.25, 2.50, 2.75, 3.00, 3.25, 3.50, 4.00, 6.00, 8.00, 10.00, 12.00, 16.00, and 24.00 h after drug administration. All the samples were centrifuged in a refrigerated centrifuge at 4°C with a rate of 3000 rpm for 10 min, as soon as possible to separate the plasma. The statistical analysis was performed on the pharmacokinetic parameters of the carbocisteine capsule using SAS^®^, statistical software Version 9.2; SAS Institute Inc., USA. The study was initiated after the appropriate ethics committee’s approval of the protocol and the informed consent documents.

The primary objective for this study was to demonstrate the bioequivalence between two carbocisteine formulations in 36 healthy, adult, human subjects under fasting conditions on the basis of the pharmacokinetic (PK) parameters: C_max_, AUC_0-t_, AUC_0-∞_ The secondary objective was to monitor the safety of subjects and to assess other pharmacokinetic data on the basis of PK parameters: T_max_, t_1/2_, K_el._

Subjects were housed (check-in) in the clinical unit of early development at least 12 h before drug administration until the 24^th^ h blood sample was drawn (check-out). Following an overnight fast of at least 10 h, a single dose of either the test or reference (carbocisteine) was administered with 240 mL of drinking water, during each period of the study under the supervision of trained study personnel. During the course of the study, the safety parameters assessed were vital signs (blood pressure, pulse rate, oral body temperature) performed at 1.00, 3.00, 6.00, 12.00, and 24.00 h post-dose in each period and also as part of the physical examination at the end of both of the periods. The haematology and biochemistry investigations done at the screening were repeated at the end in the last blood draw of period II.

**Fig. 3.**
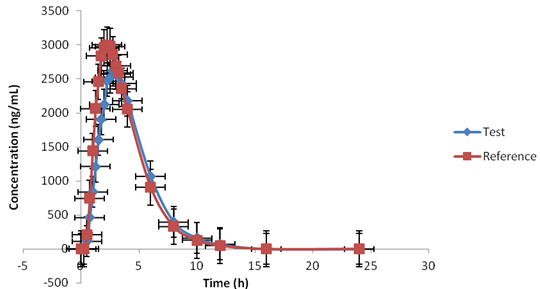
Graph of the mean plasma carbocisteine concentration (ng/mL) vs. time (h)

The 90% confidence intervals for l_n_C_max_ ranges from 82.28 to 99.18, for l_n_AUC_0-24_ ranges from 89.93 to 101.06 and for l_n_AUC_0-∞_ ranges from 90.25 to 101.29, which are within the bioequivalence range of 80–125% for carbocisteine. As the 90% confidence intervals for all the primary parameters lie within the protocol-defined ranges of 80–125%, we can conclude that the test and reference are bioequivalent. Descriptive statistics of the pharmacokinetic parameters for the test (Table 9) and reference (Table 10) and the results of the mean plasma (Figure 3) graph are given below.

**Tab. 9.** Descriptive statistics of the pharmacokinetic parameters for the test

Variable	N	Mean	SD	Min	Max	CV (%)
C_max_ (ng/mL)	35	3151.55	904.24	1789.41	5095.8	28.69
AUC_0-24_ (ng × h/mL)	35	12173.02	2474.44	7193.54	17102.42	20.33
AUC_0-∞_ (ng × h/mL)	35	12362.76	2481.19	7331.91	17327.54	20.07
T_max_ (h)	35	2.88	0.97	1.25	6.00	33.86
t_1/2_ (h)	35	1.59	0.17	1.30	2.05	10.61
K_el_ (h)	35	0.44	0.04	0.34	0.53	10.12

**Tab. 10.** Descriptive statistics of the pharmacokinetic parameters for the reference

Variable	N	Mean	SD	Min	Max	CV (%)
C_max_ (ng/mL)	35	3516.28	1049.11	1315.46	5985.56	29.84
AUC_0-24_ (ng × h/mL)	35	12909.00	3100.44	5356.92	21130.54	24.02
AUC_0-∞_ (ng × h/mL)	35	13073.20	3123.70	5472.90	21370.52	23.89
T_max_ (h)	35	2.40	0.78	1.25	4.00	32.47
t_1/2_ (h)	35	1.52	0.12	1.27	1.74	7.76
K_el_ (h)	35	0.46	0.04	0.40	0.54	7.86

## Conclusion

In this paper, a simple, sensitive, and selective LC-MS/MS method was developed and validated for the quantification of carbocisteine in human plasma as per FDA regulatory guidelines. Good recovery was observed in plasma by using the protein precipitation extraction method. The method provided good linearity in a concentration curve range of 50.000 ng/mL to 6000.000 ng/mL. To the best of our knowledge, this is the first sensitive method reported on carbocisteine in human plasma. The overall run time was 4.50 min. This method was found suitable to analyze human plasma samples for pharmacokinetic and BA/BE studies.
